# Effects of Feeding a Hypoallergenic Diet with a Nutraceutical on Fecal Dysbiosis Index and Clinical Manifestations of Canine Atopic Dermatitis

**DOI:** 10.3390/ani11102985

**Published:** 2021-10-16

**Authors:** Eleonora Elisa Alice Guidi, Alessandro Gramenzi, Paola Persico, Roberta Di Prinzio, Daniele Di Simone, Luisa Cornegliani

**Affiliations:** 1 “Città di Torino” Veterinary Clinic, 10135 Turin, Italy; 2 Veterinary Teaching Hospital, Faculty of Veterinary Medicine, University of Teramo, 64100 Teramo, Italy; agramenzi@unite.it; 3 Freelance Veterinarian, 20151 Milan, Italy; mp.persico@virgilio.it; 4 Faculty of Veterinary Medicine, University of Teramo, 64100 Teramo, Italy; rdiprinzio@unite.it; 5 Visiting Researcher CIAT, 00135 Rome, Italy; daniele.disimone@uniba.it; 6 “S. Siro” Veterinary Clinic, 20151 Milan, Italy; l.cornegliani@clinvetorino.eu

**Keywords:** atopic dermatitis, gut microbiota, skin, nutraceutical, dog

## Abstract

**Simple Summary:**

The prevalence of canine atopic dermatitis in developed countries has been growing constantly over the last few decades. Genetic predisposition represents only part of the problem, and environmental factors are believed to be an important boost to the rapid rise in atopic dogs. Although a complete understanding of the gut–skin axis has not yet been achieved, a growing number of studies demonstrate a close relationship between gastrointestinal imbalance and skin diseases. The aim of this study was to assess the effect of a nutraceutical product on the Dysbiotic Index and skin lesions of atopic dogs.

**Abstract:**

Background: an imbalance of the intestinal microbiota can cause health problems in the gastrointestinal tract and in other organs. Canine Atopic Dermatitis (CAD) is a genetically predisposed, inflammatory and pruritic allergic skin disease with multifactorial etiology and multimodal treatment. The aim of this study was to assess the effect of a nutraceutical product on Dysbiotic Index (DI) and the skin lesions of atopic dogs. Methods: a nutraceutical product was administered to 32 dogs with CAD. The product was associated with a standardized hypoallergenic diet for 60 days; the dietary regimen continued for 120 days, while ongoing therapies remained unchanged. Values of Visual Analogic Scale (VAS), Canine Atopic Dermatitis Lesional Index (CADLI) and DI were evaluated on day 0, 60, 120. Results: all the 32 dogs showed a statistically significant decrease (*p* < 0.001) to V60 of VAS and CADLI, which persisted and increased to V120 when diet alone was continued. The decrease in the DI value was also statistically significant (*p* < 0.001). Conclusion: the intake of nutraceutical associated with diet resulted in a decrease in the index of intestinal dysbiosis, with an improvement in the subjective severity of cutaneous lesions.

## 1. Introduction

Canine atopic dermatitis (CAD) is a genetically predisposed, inflammatory and pruritic allergic skin disease with specific clinical features and is commonly associated with the production of IgE antibodies to environmental allergens [1]. Diagnosis is achieved through the exclusion of all other pruritic diseases with overlapping clinical signs [2]. There may be a close relationship between CAD and adverse food reactions (AFR) [3], which include both immune-mediated and non-immune-mediated reactions and show similar gastrointestinal and cutaneous clinical signs. The therapeutic choice is determined by the patient’s condition and the severity of the disease. CAD usually requires a multimodal therapeutic approach that can include: allergen-specific immunotherapy, antipruritic drugs (Janus Kinase Inhibitors, calcineurin inhibitors) and restructuring agents for skin barrier and its microbiota (nutraceuticals and phytotherapeutics) [4]. Multimodal therapy in the long-term management of CAD has historically included the use of polyunsaturated fatty acids, in particular Omega 3 and Omega 6 [5,6,7,8,9]. These have been shown to be useful in decreasing the clinical signs of the disease, both in oral and topical administration. The use of blackcurrant seed oil has been the subject of numerous investigations with encouraging results [10,11,12]. Some studies have led to a correlation between the good condition of the skin with a healthy gastrointestinal system. The resident gut microbiota has many important functions: regulation of the immune system, maintenance of structure and function of the intestinal barrier, and protection against invasion of enteropathogens and it also provides significant nutritional benefits for the host [13]. Its complexity has been analyzed by phylogenetic, molecular and metagenomic studies that have revealed a highly diverse microbial community [12]. An imbalance of the intestinal microbiota (dysbiosis) has been associated with serious diseases not only in the gastrointestinal tract, but also in other organs. In veterinary medicine, significant differences between the fecal microbiome of healthy dogs and the microbiome of dogs with chronic gastrointestinal disorders have been identified [14,15]. In developed countries, changes in diet and overuse of antibiotics have impaired microbiota resilience and diversity; this phenomenon may account for some of the dramatic rise in autoimmune and inflammatory disorders seen both in humans and in animals [16,17]. The microbiome has an important function in educating the immune system and biodiversity is needed to achieve tolerance and to avoid allergy [18]. As in humans, hallmarks of canine AD are an abnormal immune response to environmental allergens and an impaired epidermal barrier [19]. Differences in fecal microbiota have been demonstrated in human medicine in patients with atopic dermatitis compared to healthy subjects. Patient with AD had less *Bifidobacterium*-forming colony units compared to healthy controls and occurrence of *Staphylococcus* spp. was higher in affected subjects [20,21]. Small intestinal bacterial overgrowth is 10 times more common in patients with dermatologic disorders than in healthy controls [19]. Marsella et al. demonstrated that early supplementation with probiotics led to lower values of allergen-specific IgE and partially prevents AD [22]. Research has demonstrated that treating small intestinal bacterial overgrowth (SIBO) led to a marked clinical improvement of concurrent skin disease [19] and administration of probiotics seemed to reduce or prevent clinical signs of AD in half of 13 randomized placebo-controlled trials [23]. As a consequence, intestinal dysbiosis may induce a significant worsening of allergic skin diseases. Administration of probiotics could be beneficial in preventing or reducing the clinical signs (and subsequent use of drugs) in dogs with AD [5].

The aims of the present study are: (I) to assess whether the daily administration of a nutraceuticals, consisting of blackcurrant seed oil, heat-killed *Lactobacillus reuteri*, zinc oxide and nucleotides (Ribes Pet Symbio™, NBF Lanes, Milan, Italy) can improve skin conditions in atopic dogs on an hypoallergenic diet associated or not with systemic treatment with lokivetmab or oclacitinib; (II) to evaluate the composition of the gut microbiota and dysbiosis before and after therapy and 60 days after its discontinuation.

## 2. Materials and Methods

### 2.1. Selection of Animals

The present investigation was carried out as a case-control study and each patient was its own control. The 45 animals that participated in the study were recruited by the investigators in their daily clinical practice; they are between 2 and 12 years of age, of different breeds and sexes, with a body weight greater than 1 kg and smaller than 70 kg (Table 1). The dogs enrolled were on maintenance therapy for AD; AFR (allergic food reaction) and was previously excluded by a commercial hydrolyzed diet trial in all the patients before the diagnosis and treatment of AD. Dogs were on monthly antiparasitic treatment for the control of ecto-endoparasites. Ongoing maintenance therapies included: lokivetmab (1 mg/kg/sc/monthly), specific immunotherapy for at least one year, and oclacitinib (0.4–0.6 mg/kg/os/q24 h) for at least 3 months. The animals included were free from other diseases of an immune or endocrine origin; they were also free from bacterial, fungal and parasitic infections. Dogs that had taken antibiotics, antimycotics or corticosteroids in the 30 days preceding the inclusion visit were excluded from the study. During the clinical trial, the use of other fatty acid-based, aliamides-based or other supplements was not allowed. The animals whose owners did not correctly perform the treatments did not attend the control visits; those who deviated from the protocol as well as the dogs that had to receive antibiotics or antimycotics systemically for a duration of more than 10 days or that contracted endo/ectoparasitosis during the trial were also excluded from the investigation. Furthermore, the dogs that experienced adverse treatment effects, such as erythema, pruritus, or diarrhea, for more than 10 days, or constipation and straining in defecation, were also eliminated from the trial.

### 2.2. Study Design

The study was divided into 2 phases:

Phase I. During this phase all dogs included in the trial were given an exclusive dietary regimen of hydrolyzed chicken and rice (Hypoallergenic Royal Canin ™, France) in adequate amounts, as reported by the manufacturer. The diet was associated with daily administration of the nutraceuticals studied (Ribes Pet Symbio ™, NBF Lanes Milan, Italy) at a dose of 1 capsule per 10 kg live weight once a day (Table 2). During Phase I, 3 visits were carried out: inclusion visit (day 0), on day 30 and day 60. On day 0, VAS (Visual Analogic Scale) and FeSc (Fecal Score) assessed by the owner as well as a detailed medical record with CADLI (Canine Atopic Dermatitis Lesion index) rated by the investigator, were completed for each dog included in the study. VAS and CADLI are the two most common and solid methods used to assess severity of CAD manifestations, evaluating respectively pruritus and lesions [24,25,26]. A stool sample was also requested before the trial started. The follow-up visits were set at day 30, 60 and both diet product and supplement under study were provided at each visit. At each follow-up visit, the medical record was updated and CADLI, VAS and fecal scores were noted. In addition, each owner was asked to deliver a stool sample collected within 12 h before the visit. Any adverse reactions or changes in treatment were also reported.

**Figure 1:**
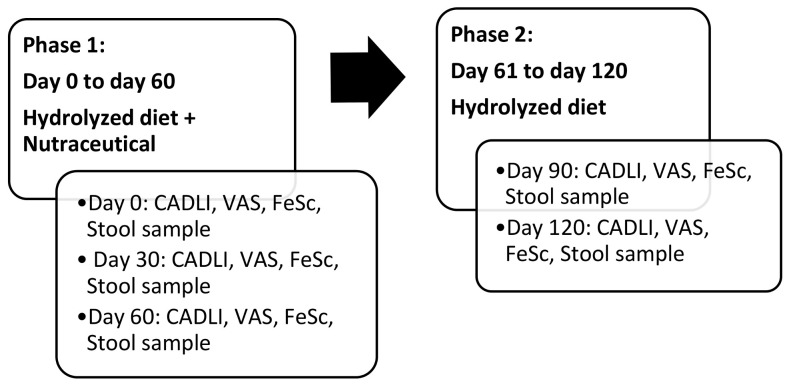
Study design flow chart.

Phase II. After day 60, all animals continued the dietary regimen alone for another two months. The follow-up visits were set at day 90 and 120 and at the same time the medical record was updated and CADLI, VAS and fecal scores were stated. At each follow-up visit, the hydrolyzed formula was provided, and the owner delivered a stool sample collected within 12 h before the visit. Any adverse reactions or changes in treatment were marked (Figure 1).

### 2.3. Evaluation of Stool and Fecal Microbiome

Fecal samples were provided by the owner at time 0, 30, 60, 90 and 120; feces did not exceed half of the container, nor were they less than 1:10 of the specific container. The stools were then stored at a temperature of −20 °C until shipment. After the conclusion of the study, samples were shipped by courier under controlled refrigerated temperature. The feces previously collected and stored were analyzed using a flotation test, coproantigen test for endoparasites, Giardia antigen (IDEXX SNAP^®^ Giardia test) and PCR for the net-F toxin gene encoding of *C. perfringens*. The degree of dysbiosis was quantified as a single numerical value, called the dysbiosis index (DI). This is a quantitative value based on 7 bacterial taxa: *Faecalibacterium*, *Turicibacter*, *Streptococcus*, *E. coli*, *Blautia*, *Fusobacterium* and *C. hiranosis*. A DI value less than or equal to 0 must be interpreted as normobiosis; the higher the DI, the more the sample is considered to deviate from normobiosis [27].

### 2.4. Statistical Analysis

To analyze the available data, a mixed effects linear model was used to evaluate variations of the parameters over time (http://biometria.univr.it/sesm/files/numeroscamp.PDF). A *p* value < 0.05 was considered statistically significative. The following independent variables were considered in the study: CADLI, VAS, Fecal Score, Dysbiosis index, Universal (sum of the seven bacterial taxa analyzed), *Faecalibacterium*, *Turicibacter*, *Streptococcus*, *E. coli*, *Blautia*, *Fusobacterium* and *C. hiranosis* (Table S1). The trend of the independent variables was corrected based on the control variables. The following regressors have been included in the model: 1. Days: continuous variable: referring to the number of days elapsed (0, 30, 60, 90, 120); 2. Gender: binary variable: 1 if the subject is male, 0 if the subject is female; 3. Weight: continuous variable: subject weight index; 4. Neutering/Sterilization: binary variable: 1 if the subject has been neutered/sterilized, 0 otherwise.

## 3. Results

Only 32 dogs out of the 45 animals recruited for the study completed the trial. Four subjects withdrew from the study as the owners failed to administer the diet and/or the nutraceutical. The dogs commonly refused to assume the diet or the nutraceutical as prescribed. Six dropped out due to the onset of other pathology and consequent administration of antimicrobial or anti-inflammatory treatments. The remaining 3 patients were excluded as they were not compliant to follow-up visits. The mean age of the selected group was 8,1 years (range: 2–9); there were 15 female and 7 neutered female, 9 male and 1 neutered male. Breeds, weight and previous diet are reported in Table 1. During the 4 months of the study, all the animals continued the medical therapy for the control of pruritus as previously established. Flotation test, coproantigen test for endoparasites, Giardia antigen and PCR for the net-F toxin gene coding of *C. perfringens* were negative in all the samples collected. An initial sample size calculation assuming a probability (power) of 0.99 and an alpha of 0.05 suggested studying forty-five experimental subjects. Thirty-two dogs completed the trial, so the power dropped to 0.97, maintaining an alpha of 0.05 (htpp://biometria.univer.it/sesm/files/numeroscamp.PDF). By analyzing patient data on day 0, 30 and 60, statistically significant differences have been detected for all clinical parameters considered (VAS, CADLI and Fecal Score).

### 3.1. VAS, CADLI and FeSc 

**Figure 2:**
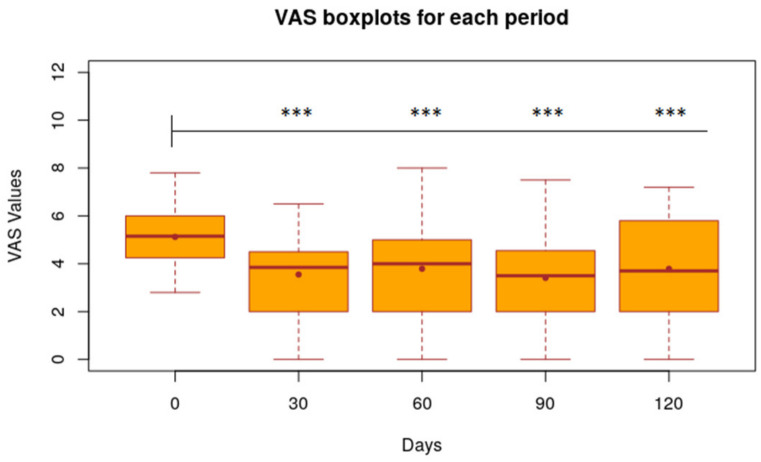
Statistical results of VAS values calculated for each period. Boxplot of VAS value of 32 dogs at time 0, 30, 60, 90, 120 days. Level of Significance compared to time 0: *** 1%; ** 5%; * 10%.

Taking day 0 as a reference, the average VAS was 1.57 points lower (*p* < 0.001) on day 30 and 1.33 points (*p* < 0.001) on day 60 (Figure 2).

**Figure 3:**
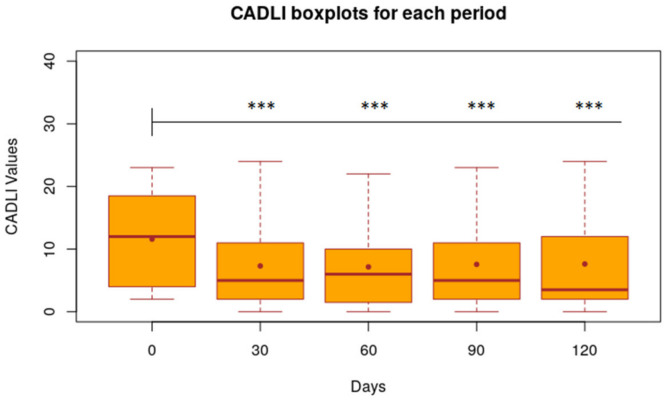
Statistical results of CADLI values calculated for each period, Boxplot of CADLI value of 32 dogs at time 0, 30, 60, 90, 120 days. Level of Significance compared to time 0: *** 1%; ** 5%; * 10%.

CADLI decreased by 4.28 points (*p* < 0.001) on day 30 and 4.43 points (*p* < 0.001) on day 60 (Figure 3).

**Figure 4:**
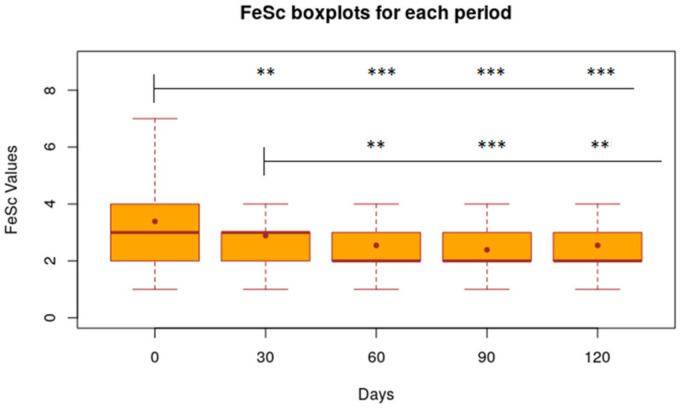
Statistical results of FeSc values calculated for each period. Boxplot of FeSc value of 32 dogs at time 0, 30, 60, 90, 120 days. Level of Significance compared to time 0 and 30 days: *** 1%; ** 5%; * 10%.

FeSc was significantly reduced by 0.50 points (*p* < 0.005) on day 30 and 0.84 points (*p* < 0.001) on day 60 (Figure 4). In the interval between 30 and 60 days, the only significantly decreased parameter is the Fecal Score (−0.34 with *p* < 0.01).

After the nutraceutical administration was stopped (day 60), the average values of VAS, CADLI and Fecal Score showed no significant differences in the time intervals between day 60 and 90, between day 60 and 120 and between day 90 and 120. Figure 2, Figure 3 and Figure 4 show the results of the statistical analysis, which were conducted on VAS, CADLI, and FeSc values during the different time points of the study.

### 3.2. Medical Therapy

By classifying patients based on medical therapy for the control of pruritus, 4 groups can be identified: no systemic therapy (3 dogs), specific immunotherapy (7 dogs), lokivetmab (6 dogs) and oclacitinib (16 dogs) (Table 3). If compared to the group of patients without treatment, drug administration makes a significant contribution only for the VAS parameter: in the lokivetmab group, VAS average value at day 60 is −18.37 (*p* < 0.01); in the oclacitinib group, VAS mean value is −9.28 (*p* < 0.05); in the group treated with immunotherapy, no statistically significant results were found.

### 3.3. Dysbiosis Index

**Figure 5:**
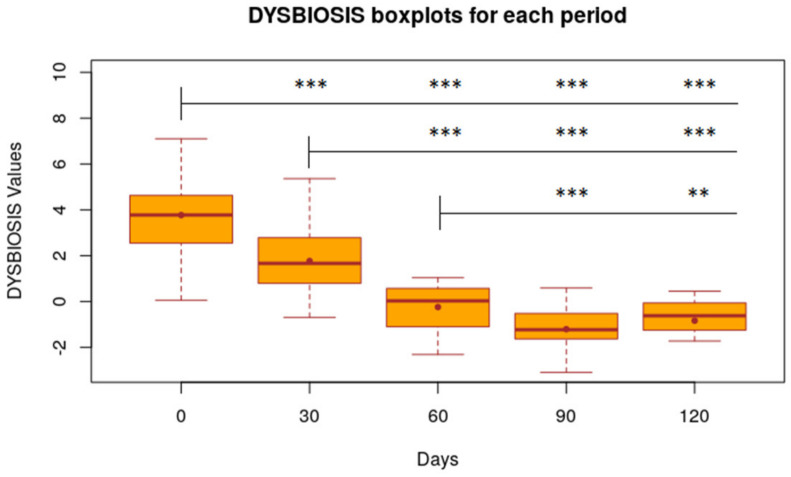
Statistical results of dysbiosis index calculated for each period Boxplot of FeSc value of 32 dogs at time 0, 30, 60, 90, 120 days. Level of Significance compared to time 0, 30 and 60 days: *** 1%; ** 5%; * 10%.

The dysbiosis index measured in the period from day 0 to day 30, starting from T0 as a reference (DI T0 = 3.81), decreased by 2.00 points (*p* < 0.001); from day 0 to day 60 this value turned out to be further reduced: −4.01 (*p* < 0.001) (Figure 5). The decrease in the DI value is statistically significant also between day 30 and 60: −2.01 (*p* < 0.001). The dysbiosis index shows a downward trend, even after discontinuation of treatment with the nutraceutical; in fact, from day 60 to 90, the DI decreased by 0.96 points (*p* < 0.01) and from day 60 to day 120 it continued to decrease by 0.62 (*p* < 0.01). The DI from day 90 to day 120 increased by 0.36 points, although this figure is not statistically significant (Figure 5). Values of the dysbiosis index in patients grouped according to drug therapy yielded no statistical relevance.

## 4. Discussion

The results of this study demonstrate a decrease in VAS and CADLI values during nutraceutical administration and a hydrolyzed diet. Pruritus and lesion scores did not improve further between days 60 and 120 (only with dietary regimen) and, also if not statistically significant, tended to return to the initial values (D0). The FeSC values instead remained statistically unchanged throughout the duration of the study. It should be emphasized that, however, all the dogs included were already on a controlled diet with commercial dietary regimen and/or protein hydrolysates (Table 1). It is not unusual to have owners that prefer to maintain their pets under a “hypoallergenic diet” instead of changing to commercial ones. In ours cases, the owners of selected dogs had preferred to return to a dietary controlled regimen after a negative trial test for FIAD (Food Induced Atopic Dermatitis).

Assuming that a dysbiosis index less than or equal to zero represents an eubiotic system, the results obtained show that, starting from day 0 as a reference, the dysbiosis index decreased from day 0 to day 30, and on day 60 this value was further reduced [27]. Unlike the clinical parameters, the dysbiosis index shows a decreasing trend following the discontinuation of treatment, with the nutraceutical between days 60 and 90. The modulation of the microbiota by the product seems to persist for another 30 days after the discontinuation. However, although the DI appears to have decreased on day 120 by 0.62 (*p* <0.01) as opposed to day 60, the value tends to increase if compared to day 90. The DI from day 90 to day 120 increases by 0.36 points, although this increase is not statistically significant; this data point shows a progressive tendency of the microbiota to return to the initial values before inclusion in the study. Resilience of the microbiota itself, however, especially in long-term dysbiotic subjects, might justify the tendency to increase, as shown by the DI value [28].

The mechanisms involved in the manifestation of atopic dermatitis are complex and little is known about the role of gut microbiota in the pathogenesis of AD. However, the intestinal microbiota could play a crucial role by regulating the maturation of the immune system through cross-communication between microbiota and host, especially at a young age [29,30]. The gut microbiota has an enormous ability to synthetize molecules, alterations in the composition of intestinal microbial populations may positively or negatively influence health through the production of metabolites and the activation of pro-inflammatory cells [12]. In recent years, there has been a lot of interest in the inclusion of pro/prebiotics and paraprobiotic in the canine diet or nutraceuticals [31,32,33,34].

In this study, a tyndalized *Lactobacillus reuteri* was present in the nutraceutical tested. Tyndalized bacteria have been subjected to a heat treatment that rendered it unable to metabolize and reproduce. Their contribution constitutes a suitable substrate in the intestine for the recolonization of the symbiotic microbiota and is reconstituted and at the same time as there is a contrast in the establishment and development of all pathogenic or harmful strains. On a physiological level, they perform the same effects as the use of classic “live” probiotics, such as improved intestinal function, stimulation of the immune system, and an increase in the general state of well-being. In recent years, the term “paraprobiotic” has been coined for tyndalized bacteria to define inactivated probiotics as non-viable microbial cells or crude cell extracts (e.g., with complex chemical composition), which, if administered in adequate quantities, confer a benefit to the animal organism [35].

The optimization of nutrient intake, intestinal microbiota and its immune function could represent an excellent strategy to promote the beneficial effects on health in general, including the decrease of clinical signs of allergic skin diseases. In fact, the dogs included in the study, albeit with a stable dermatological disease, all showed a reduction in VAS and an absence of recurrences, as well as a decrease in intestinal dysbiosis during treatment with the nutraceutical. It would be interesting to evaluate whether the regular and prolonged intake over time of pro- and prebiotics may decrease the incidence of inflammatory diseases in dogs. Previous studies in human medicine have shown that the gut microbiota in early childhood is associated with age of onset, severity, remission, exacerbation and even phenotypes of AD [36].

Conventional antipruritic and antimicrobial therapies used for the control of the disease and its recurrences may exacerbate such factors as intestinal permeability and dysbiosis, promoting the development of antibiotic-resistant microbes [37]. Yet, the findings from this study have shown that ongoing therapies with lokivetmab, specific immunotherapy and oclacitinib do not affect FeSc and DI values. The patients included in the trial had no ongoing antimicrobial drug treatment, at least from 1 month. In this regard, it would be interesting to be able to assess whether the use of this nutraceutical may help restore a healthy gut microbiota, even in the presence of antibiotic or/and antimycotic therapies.

The mechanisms of gut–skin communication have not yet been fully understood, although numerous studies in human medicine have shown that many dermatoses, including atopic dermatitis, appear to be linked to gut health [36]. In humans, it has been proven that intestinal dysbiosis causes an increase in intestinal barrier permeability and a decrease in the production of short-chain fatty acids. The increased permeability allows the translocation of metabolites and bacterial DNA to the blood stream and consequently to the skin, where they cause activation of keratinocytes and T-cells. The subsequent immune and metabolic response induces skin microbiota dysbiosis [38]. An intestinal microbiome can affect skin the microbiome also through neuroendocrine signaling: tryptophan produced by microbes in the gut stimulates an itch in humans with atopic dermatitis [39]. In veterinary medicine, the presence of FIAD reveals a correlation between these two entities [3]. It follows that gut dysbiosis and increased intestinal permeability may induce a significant worsening in allergic skin diseases, but all the possible pathways need further research. The present study highlighted how dogs with CAD have intestinal dysbiosis.

## 5. Conclusions

The evaluation of the effects of treatment with the nutraceutical appears to reveal a contribution to modulate the canine intestinal microbiota, thus leading to a change in the dysbiotic conditions found in atopic dogs. Regardless of the ongoing therapies and the best quality of industrial food, the animals included in the trial are dysbiotic dogs [40]. Further studies are needed to assess the potential positive effects of nutraceutical intake for more extended periods of time in these patients.

## Figures and Tables

**Table 1:** Dogs with atopic dermatitis that finished the study.

Breed	Age	Gender	Weight	Therapy	Previous Diet
Abruzzese Maremma shepherd dog	8	nf	45	immunotherapy	hydrolised diet (Z/D Hill’s)
French bulldog	3	nf	10	immunotherapy	hydrolised diet (Anallergenic, Royal Canin)
French bulldog	4.5	f	15.5	immunotherapy	monoproteic diet (Exclusion, Pork)
English bulldog	4	m	31	immunotherapy	hydrolised diet (Trainer, Rabbit)
Labrador retriever	2.5	f	40	immunotherapy	hydrolised diet (Z/D Hill’s)
Mongrel	6	nf	38.5	immunotherapy	hydrolised diet (Z/D Hill’s)
Newfoundland	5	m	68	immunotherapy	monoproteic diet (Forza 10, Beef)
Labrador retriever	2	f	25	lokivetmab	hydrolised diet (Hypoallergenic Royal Canin)
Border collie	4	f	24	lokivetmab	monoproteic diet (Trainer, Beef)
Boxer	2	m	20	lokivetmab	monoproteic diet (Exclusion, Rabbit)
French bulldog	2	f	10	lokivetmab	monoproteic diet (Exclusion, Salmon)
German shepherd	7	f	37	lokivetmab	hydrolised diet (H/A Purina)
Chinese shar-pei	2.5	f	17	lokivetmab	hydrolised diet (H/A Purina)
American Stafforshire terrier	2	m	35	none	monoproteic diet (Trainer, Pork)
English bulldog	2	m	28	none	monoproteic diet (Trainer, Rabbit)
Mongrel	6	f	18.5	none	limited-antigen diet (Acana, Pork and Beef)
Border collie	8	m	21	oclacitinib	hydrolised diet (Z/D Hill’s)
Boxer	3.5	f	23.8	oclacitinib	hydrolised diet (Hypoallergenic Royal Canin)
Cane corso	8	nf	54	oclacitinib	hydrolised diet (H/A Purina)
Dogue de Bordeaux	6	nf	62	oclacitinib	monoproteic diet (Trainer, Rabbit)
Dogue de Bordeaux	2	m	52	oclacitinib	monoproteic diet (Forza 10, Beef)
Golden retriever	4.7	f	33	oclacitinib	hydrolised diet (Z/D Hill’s)
Golden retriever	4	m	35	oclacitinib	monoproteic diet (Exclusion, Pork)
Jack Russel terrier	2.8	f	8.1	oclacitinib	monoproteic diet (Trainer, Pork)
Maltese	2.3	f	5	oclacitinib	monoproteic diet (Prolife, Pork)
Mongrel	8	nm	24	oclacitinib	monoproteic diet (Exclusion, Pork)
Mongrel	5	nf	20	oclacitinib	monoproteic diet (Prolife, Beef)
Mongrel	9	nf	14	oclacitinib	monoproteic diet (Prolife, Rabbit)
Chinese shar-pei	2.5	f	14.5	oclacitinib	fatty acid-enriched diet (Derm Defence Hill’s)
West Higland white terrier	9	f	8.3	oclacitinib	limited-antigen diet (Sensitivity Royal Canin)
West Higland white terrier	8	f	8.9	oclacitinib	limited-antigen diet (Sensitivity Royal Canin)
West Higland white terrier	2	m	11	oclacitinib	hydrolised diet (Anallergenic Royal Canin)

Legend: f: female; nf: neutered female; m: male; nm: neutered male.

**Table 2:** Ribes Pet Symbio composition.

Components	Dose
Black currant seed oil	625 mg/capsule
Heat-inactivated *Lactobacillus reuteri* NBF 1	200 mg/capsule
Zinc oxide	25 mg/capsule
Nucleotides	50 mg/capsule

**Table 3:** Ongoing treatment at day 0 and at the end of the clinical study.

Dogs	Therapy	Composition of Immunotherapy	Dose
7	Specific immunotherapy	2 DF, TP; 4 DF, TP, AS; 1 DF	Maintenance therapy 1 mL/sc once a month
6	Lokivetmab		1 mg/kg/sc once a month
3	None		
16	Oclacitinib		0.4–0.6 mg/kg/q24 h orally

Legend: DF Dermatophagoides farinae; TP Thyrophagus putrescentie; AS Acarus siro.

## Supplementary Materials

The following are available online at https://www.mdpi.com/article/10.3390/ani11102985/s1, Table S1: Abundance of bacterial taxa and calculated Dysbiosis Index of dogs at different study days.
