# Supplementary material for: Effects of Feeding a Hypoallergenic Diet with a Nutraceutical on Fecal Dysbiosis Index and Clinical Manifestations of Canine Atopic Dermatitis
Source: Animals (Basel). 2021 Oct 16;11(10):2985. doi: 10.3390/ani11102985 (PMC8532801; doi:10.3390/ani11102985)
Supplement: Supplementary file 1 [file animals-11-02985-s001.zip › animals-1353296-supplementary.pdf]

ID	Study Day	DYSBIOSIS INDEX	log DNA total	log DNA *Faecalibacterium*	log DNA *Turicibacter*	log DNA *Streptococcus*	log DNA *E. coli*	log DNA *Blautia*	log DNA *Fusobacterium*	log DNA *C. hiranonis*
CC3	0	3,24	11,80	4,59	5,88	6,71	6,74	10,66	5,72	1,17
CC3	30	2,65	12,04	6,04	6,52	5,66	6,09	10,72	6,12	1,89
CC3	60	0,12	12,91	5,92	7,11	4,96	5,74	11,64	6,25	2,65
CC3	90	-0,35	13,92	6,33	7,47	3,88	5,39	11,76	6,68	2,89
CC3	120	-1,25	12,79	6,98	7,01	3,75	4,91	11,56	6,54	3,32
CC4	0	3,69	11,44	5,39	6,96	8,75	8,11	11,28	5,67	1,68
CC4	30	3,12	11,78	5,81	7,00	8,26	6,37	11,62	6,21	2,15
CC4	60	1,02	12,68	6,44	7,44	7,60	5,85	12,63	6,34	2,88
CC4	90	-0,23	13,55	6,62	7,62	6,52	5,54	13,31	6,12	3,45
CC4	120	-0,66	12,69	6,54	7,62	5,41	5,32	12,82	6,01	3,25
EG8	0	4,36	10,45	5,52	5,14	5,89	6,94	10,44	7,17	2,15
EG8	30	2,15	11,25	5,22	6,03	4,66	6,11	10,86	7,25	3,65
EG8	60	0,13	11,99	6,53	6,99	4,01	5,35	11,35	8,01	3,99
EG8	90	0,03	12,03	5,31	7,01	3,56	5,32	12,01	7,99	4,01
EG8	120	-0,35	12,45	6,59	6,85	3,24	5,12	11,88	7,65	3,85
ER6	0	3,64	10,87	4,33	4,63	6,44	4,53	11,56	5,34	2,56
ER6	30	2,45	11,37	4,48	5,42	5,80	4,14	11,66	5,86	3,45
ER6	60	1,32	12,19	4,33	6,45	4,54	5,62	12,26	5,87	4,70
ER6	90	-0,17	12,12	3,89	6,22	4,20	3,48	12,99	5,69	4,93
ER6	120	-1,69	12,15	4,06	6,14	3,78	3,19	13,02	5,93	4,77
ER7	0	3,59	10,64	4,84	6,78	7,96	7,04	10,45	6,20	2,86
ER7	30	3,18	11,28	5,43	6,72	6,81	6,26	11,52	6,38	3,45
ER7	60	-1,26	12,40	5,30	7,68	6,21	5,76	12,07	6,81	4,22
ER7	90	-0,54	12,66	5,71	6,02	5,13	5,26	12,14	6,60	5,12
ER7	120	-0,02	12,29	5,01	5,44	5,00	4,72	12,62	6,88	6,05
LG6	0	4,32	11,55	5,12	5,28	8,76	7,21	11,50	7,25	2,63
LG6	30	2,12	11,95	5,21	6,38	7,60	6,86	11,71	7,66	3,61
LG6	60	0,08	12,74	5,62	6,57	6,98	5,56	12,20	7,17	4,15
LG6	90	0,01	12,35	5,79	7,42	6,76	5,14	12,86	7,76	4,69
LG6	120	0,03	12,02	6,35	8,63	6,44	5,02	13,01	7,51	5,32
LG7	0	3,25	11,55	4,78	4,72	7,10	6,28	9,08	7,68	4,43
LG7	30	1,21	11,98	5,13	4,81	5,83	5,16	9,74	8,10	5,11
LG7	60	0,36	12,38	4,48	5,89	4,32	4,89	10,31	8,92	5,42
LG7	90	-0,15	13,45	4,96	6,42	3,42	4,65	11,03	8,59	5,68
LG7	120	-0,13	12,76	4,48	7,02	3,07	4,32	10,78	8,66	5,68
LG8	0	5,96	11,70	3,83	6,22	6,69	5,07	11,63	5,68	4,11
LG8	30	2,32	12,06	4,39	6,52	6,36	4,51	11,68	6,64	5,27
LG8	60	1,37	12,86	4,79	7,74	5,44	4,15	11,00	7,14	6,43
LG8	90	0,21	13,84	5,19	8,15	5,21	3,71	11,05	6,49	7,28
LG8	120	-0,01	12,65	6,34	9,14	5,09	3,51	10,65	7,87	7,28
NF5	0	5,64	11,21	3,73	5,42	7,86	5,28	10,43	5,58	4,13
NF5	30	2,32	12,03	4,19	5,72	6,60	4,16	11,01	6,54	5,03
NF5	60	1,35	12,94	4,87	6,94	6,88	3,89	,11,68	7,04	5,99

NF5	90	-0,21	12,76	5,22	7,35	5,76	3,65	12,01	6,39	6,01
NF5	120	-1,32	12,31	6,13	8,34	5,44	3,32	11,99	7,77	5,86
NF6	0	4,09	11,93	3,86	6,59	8,01	6,07	10,66	6,26	4,12
NF6	30	1,05	12,03	4,61	7,48	6,92	6,51	11,58	6,47	5,23
NF6	60	1,01	12,35	4,21	7,53	5,66	5,15	12,03	6,86	5,87
NF6	90	-1,22	12,47	3,99	8,01	4,46	4,71	12,35	6,47	6,12
NF6	120	-1,15	12,03	3,33	7,88	4,09	4,51	12,59	6,43	6,54
RS3	0	3,09	10,96	5,32	4,41	6,30	4,73	10,05	7,12	1,28
RS3	30	0,05	11,01	7,17	5,03	5,81	3,74	10,54	7,96	2,20
RS3	60	0,01	11,26	6,32	6,46	5,44	3,14	11,36	8,28	3,16
RS3	90	-0,22	11,17	5,96	7,61	4,89	2,95	11,69	8,08	3,99
RS3	120	-0,15	11,07	5,51	6,82	4,29	2,65	12,01	7,60	4,09
RS4	0	2,25	9,22	2,83	4,63	6,44	5,82	8,97	7,25	3,68
RS4	30	-1,78	11,05	6,62	5,78	5,85	4,69	10,96	7,66	4,87
RS4	60	-2,74	11,69	6,85	6,83	5,15	3,80	11,34	7,98	5,78
RS4	90	-0,44	11,53	5,96	7,61	4,28	2,90	12,03	7,95	6,20
RS4	120	-0,60	11,42	6,37	8,83	3,97	2,48	11,93	7,56	6,68
RS5	0	4,02	10,45	5,12	4,85	5,44	4,72	9,94	6,15	2,65
RS5	30	3,25	11,02	5,22	5,55	4,75	3,58	11,85	6,56	3,84
RS5	60	1,21	11,99	5,66	6,24	4,03	2,75	12,14	6,88	4,77
RS5	90	-0,03	12,01	4,88	6,89	3,56	1,99	13	6,85	5,12
RS5	120	-0,15	12,36	6,25	6,45	2,99	1,45	12,96	6,46	5,63

Supplementary Table S1. Abundance of bacterial taxa and calculated Dysbiosis Index of dogs at different study days.
